# The crystal structure of tetra­kis­(5-phenyl-1*H*-imidazole-κ*N*^3^)zinc(II) dinitrate

**DOI:** 10.1107/S2056989025010874

**Published:** 2026-01-01

**Authors:** Nomampondo Penelope Magwa

**Affiliations:** a University of South Africa, Department of Chemistry, Private Bag X6, Florida, Gauteng, 1710, South Africa; University of Missouri-Columbia, USA

**Keywords:** 5-phenyl-1*H*-imidazole, Zn-complex, crystal structure

## Abstract

The title complex salt, [Zn(C_9_H_8_N_2_)_4_](NO_3_)_2_, features a central zinc(II) ion coordinated by four 5-phenyl­imidazole ligands, with two nitrate anions providing charge balance. It crystallizes in the monoclinic space group *C2/c*. In the crystal, the nitrate ions occupy the voids formed by the [Zn(C_9_H_8_N_2_)_4_]^2+^ cations and function as counter-ions. The nitrate oxygen atoms participate in strong N—H⋯O hydrogen-bonding inter­actions. The crystal studied was refined as a two-component twin.

## Abstract 2

In the title compound, the central zinc(II) ion coordinated by four 5-phenyl­imidazole ligands, with two nitrate anions providing charge balance·In the crystal, the nitrate ions occupy the voids formed by the [Zn(C_9_H_8_N_2_)_4_]^2+^ cations and function as counter-ions. The nitrate oxygen atoms participate in weak N—H⋯O hydrogen-bonding inter­actions.

## 1. Chemical context

The 5-phenyl-1*H*-imidazole scaffold is an important framework in medicinal chemistry due to its versatility and biological significance. It significantly contributes to the creation of pharmacologically active mol­ecules, particularly in the fields of HIV, anti­cancer, and anti­bacterial research (Abu Almaaty *et al.*, 2021 ▸; Rashamuse *et al.*, 2020 ▸, 2021 ▸; Roy *et al.*, 2005 ▸). The imidazole moiety is frequently included in a variety of medicinal drugs, and its pharmacokinetic and pharmacodynamic qualities are further improved by the addition of a phenyl group at the 5-position (Devi *et al.*, 2024 ▸; Blass *et al.*, 2000 ▸). 5-Phenyl-1*H*-imidazole is a structurally straightforward aromatic heterocycle composed of a five-membered imidazole ring with two non-adjacent nitro­gen atoms substituted at the 5-position with a phenyl group. This basic structure has several functionalization sites, making it a promising starting point for drug discovery and synthetic modification. In addition to its biological value, imidazole derivatives, such as 5-phenyl-1*H*-imidazole, have shown great promise in coordination chemistry. These compounds readily form coordination complexes with a wide range of transition metals, including zinc, copper, ruthenium, and iron (Rashamuse *et al.*, 2023 ▸; Baranoff *et al.*, 2011 ▸; Magwa & Rashamuse, 2024 ▸; Bonomo *et al.*, 1988 ▸; Carver *et al.*, 2003 ▸; Li *et al.*, 2024 ▸). In metal complexes, the nitro­gen atom in the imidazole ring works as a σ-donor ligand, typically binding through the *sp*^2^-hybridized nitro­gen atom (also known as the imine-type nitro­gen at position 3 of the ring). This coordination stabilizes the metal center compared to its unligated or aqua-ligated state, and significantly affects the complex’s redox potential, geometry, and chemical reactivity. These metal–imidazole complexes are important in bioinorganic chemistry because they frequently serve as models for metalloenzymes (Roy *et al.*, 2005 ▸). Enzymes such as carbonic anhydrase and cytochrome c oxidase rely on imidazole moieties for catalytic activity and electron transport, respectively (Roy *et al.*, 2005 ▸; Maneeta *et al.*, 2024 ▸). Thus, synthesized imidazole–metal complexes offer important insights into enzyme functions and are being investigated for therapeutic uses including as imaging probes, anti­cancer medicines, and anti­bacterial compounds.
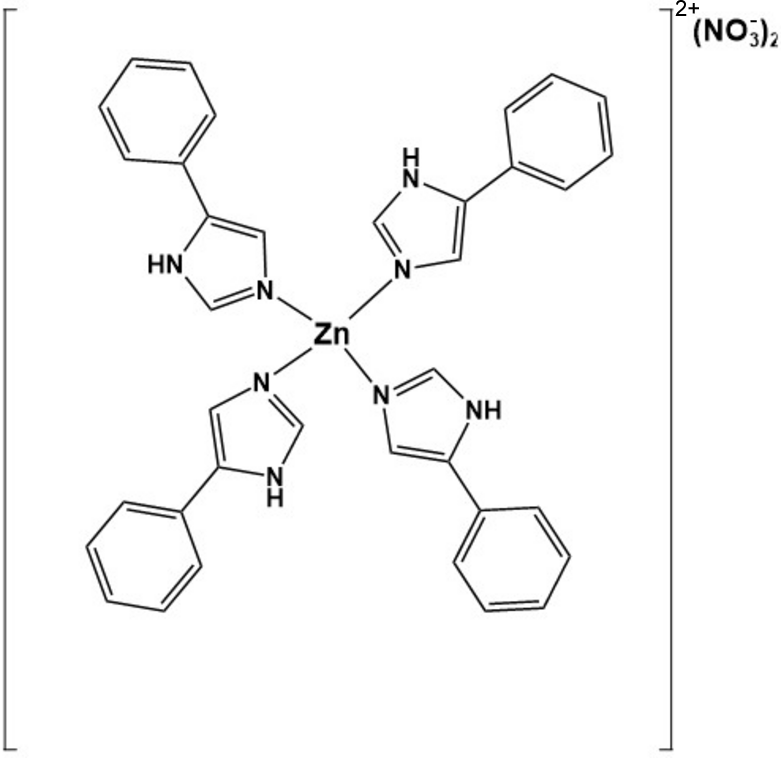


## 2. Structural commentary

**Figure 1:**
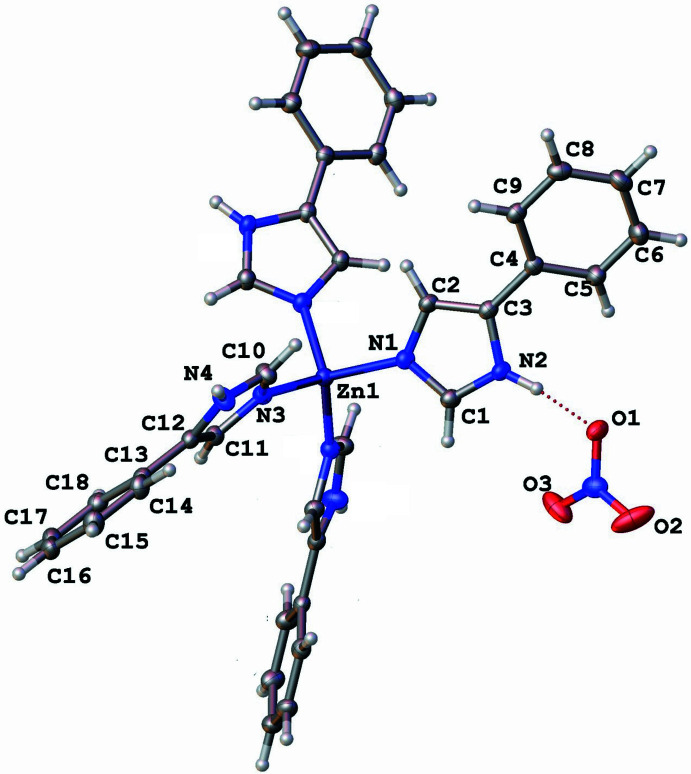
Displacement ellipsoid plot of the title compound showing the atom-numbering scheme and the inter­actions between the nitrate ion and the 5-phenyl-1*H*-imidazole ligand (dashed lines). Displacement ellipsoids are drawn at the 50% probability level. Unlabelled atoms are generated by the symmetry operation −*x* + 1, *y*, −*z* + 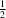.

The title compound crystallizes in the monoclinic space group *C*2/*c*, with the zinc(II) atom occupying a special position on a symmetry element. As a result, the molecule’s intrinsic symmetry matches the crystallographic symmetry, so only a fraction of the molecule is present in the asymmetric unit. The zinc(II) atom is coordinated by four 5-phenyl-1*H*-imidazole ligands, forming a distorted tetra­hedral geometry (Table 1 ▸, Fig. 1 ▸). This distortion is evident from the six N—Zn—N bond angles, which deviate slightly from the ideal tetra­hedral angle of 109.5°. The Zn—N bond lengths are consistent, with symmetry-equivalent values averaged to 1.986 Å, confirming a relatively symmetrical coordination sphere. The nitrate anions act as counter-ions and exhibit slight distortions from an ideal trigonal planar geometry (Table 1 ▸). These deviations arise from hydrogen-bonding inter­actions with the imidazole ligands, which are discussed in the *Supra­molecular features* section. Generally, the structural parameters and symmetry constraints define a stable, well-organized crystal structure, with the zinc center adopting a distorted tetra­hedral coordination environment stabilized by both covalent and non-covalent inter­actions. These interactions arise from crystal packing and intermolecular forces, which slightly adjust bond angles and distances to optimize crystal stability. As a result, the coordination sphere deviates from an ideal tetrahedron, reflecting the combined influence of both strong covalent bonding and secondary non-covalent forces.

## 3. Supra­molecular features

**Figure 2:**
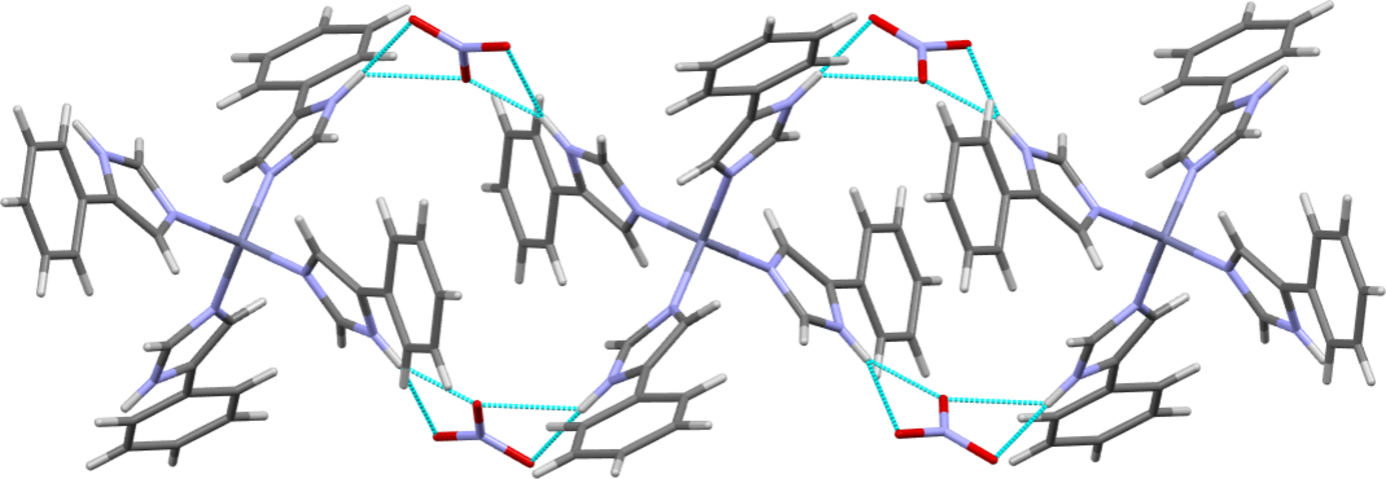
Packing diagram of the title compound showing the nitrate cations lying in the voids between the cationic complexes.

The mol­ecular packing of the title compound is illustrated in Fig. 2 ▸, showing how the three-dimensional arrangement of [Zn(C_9_H_8_N_2_)_4_]^2+^ cations and nitrate anions defines the crystal structure. The nitrate ions play an active role in shaping the packing by accepting strong N—H⋯·O hydrogen bonds (Table 2 ▸) with hydrogen atoms from the imidazole moieties. Each nitrate anion accepts three hydrogen bonds from neighboring cations, while each cation inter­acts with two nitrate anions and several adjacent cations. In addition to hydrogen bonding, phenyl rings from adjacent cations exhibit notable π–π stacking and edge-to-face inter­actions, characterized by a parallel-displaced arrangement [centroid–centroid distance = 4.037 (2) Å, interplanar separation = 3.408 Å, slippage = 2.165 Å] and a T-shaped contact [centroid–centroid distance = 5.536 (2) Å, ring-normal angle = 69.9°, centroid-to-plane separations = 3.835 and 4.137 Å], highlighting non-covalent cation–cation contacts that contribute to the crystal cohesion. Together, these hydrogen bonds and aromatic inter­actions organize the crystal into a robust structure, integrating electrostatic forces, directional hydrogen bonding, and π–π stacking. to optimize the crystal stability.

## 4. Database survey

The title complex, represents a new addition to the family of zinc(II)–imidazole derivatives, a group known for their structural flexibility and diverse coordination behavior. Zinc(II) readily adopts various geometries, while imidazole ligands provide multiple coordination possibilities. A search of the Cambridge Structural Database (CSD, Version 5.45, March 2024 update; Groom *et al.*, 2016 ▸) and Google Scholar found no prior example of a complex with the same formulation, [Zn(C_9_H_8_N_2_)_4_]^2+^·2NO_3_^−^, confirming its originality. Related zinc–imidazole complexes are rare but include Zn(C_3_H_4_N_2_)_4_^2+^ (CCDC No. 639568; Huang *et al.*, 2007 ▸), [Zn(dmit)_4_][BF_4_]_2_, and [Zn(dmit)_4_][NO_3_]_2_ (CCDC Nos. 772715 and 772716; William *et al.*, 2010 ▸), helical frameworks [Zn(bdt)]^2+^ (CCDC Nos. 772872 and 772873; Liu *et al.*, 2010 ▸), and Zn(C_4_H_6_N_2_)_4_^2+^ (CDCC No. 861722; Reedijk *et al.*, 2012 ▸). Structural comparison with these reported systems shows that Zn—N bond lengths in tetra­hedral Zn^II^ complexes generally range from 1.97–2.00 Å, consistent with the mean value of 1.986 Å in the present compound. Likewise, the observed N—Zn—N bond angles [105.44 (6)–111.70 (4)°] fall within the expected range of 104–113° reported for similar complexes in the CSD, confirming a slightly distorted tetra­hedral environment. Such deviations from the ideal tetra­hedral angle of 109.5° are common and have been attributed to steric effects of bulky substituents and hydrogen-bonding inter­actions (Huang *et al.*, 2007 ▸; William *et al.*, 2010 ▸; Reedijk *et al.*, 2012 ▸). In this complex, the distortion likely arises from the phenyl-imidazole ligands and nitrate-mediated hydrogen bonding, in agreement with established structural trends for imidazole-based Zn^II^ systems.

## 5. Synthesis and crystallization

To prepare the title compound, 0.14 g (0.485 mmol) of zinc nitrate hexa­hydrate were added to a stirred solution of 0.58 g (4 mmol) of 4-phenyl­imidazole in a solvent mixture of di­chloro­methane (DCM, 25 mL) and methanol (MeOH, 3 mL) in a round-bottom flask. The resulting slurry was stirred continuously for 5 h until a clear solution formed, after which the solution was filtered, and the filtrate was allowed to evaporate slowly at ambient temperatures (298–300 K). After 16 days, light-yellow crystals formed, which were collected by filtration and dried in air. The synthesis is shown in the scheme below.
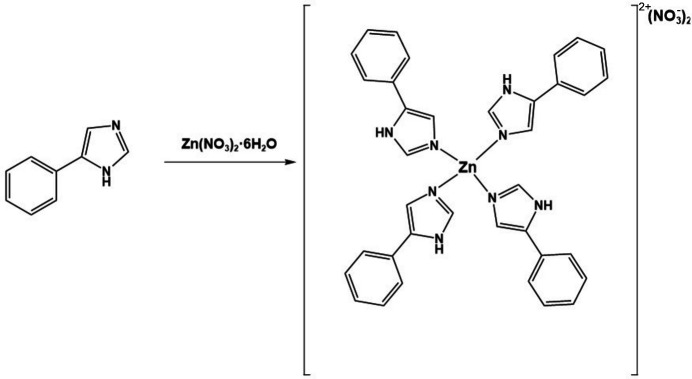


### 6. Refinement

Crystal data, data collection and structure refinement details are summarized in Table 3 ▸. All C-bound H were placed in geometrically idealized positions and refined using the riding model, with isotropic displacement parameters set to 1.2 or 1.5 times those of the corresponding parent carbon atoms. The crystal studied was refined as a two-component twin, and an appropriate twin law was applied during the refinement process. A B-level PLAT910 alert indicates that low-angle reflections below θ_min_ = 3.92° were omitted. This exclusion is intentional because the reflections at such low angles were either partially obscured by the beamstop or detector gap or severely overloaded. The omission prevents systematic errors in intensity data without affecting the completeness of the dataset or the refinement stability.

## Supplementary Material

Crystal structure: contains datablock(s) I. DOI: 10.1107/S2056989025010874/ev2022sup1.cif

Structure factors: contains datablock(s) I. DOI: 10.1107/S2056989025010874/ev2022Isup3.hkl

CCDC reference: 2433548

Additional supporting information:  crystallographic information; 3D view; checkCIF report

Additional supporting information:  crystallographic information; 3D view; checkCIF report

## Figures and Tables

**Table 1:** Selected geometric parameters (Å, °)

Zn1—N1	1.9882 (11)	O2—N5	1.2295 (19)
Zn1—N3	1.9828 (11)	O3—N5	1.2585 (18)
O1—N5	1.2606 (16)		
			
N1^i^—Zn1—N1	105.44 (6)	O2—N5—O1	119.84 (14)
N3—Zn1—N1^i^	110.19 (4)	O2—N5—O3	123.65 (14)
N3—Zn1—N1	111.70 (4)	O3—N5—O1	116.51 (13)
N3—Zn1—N3^i^	107.68 (7)		

Symmetry code: (i) 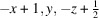.

**Table 2:** Hydrogen-bond geometry (Å, °)

*D*—H⋯*A*	*D*—H	H⋯*A*	*D*⋯*A*	*D*—H⋯*A*
N2—H2⋯O1	0.88	1.91	2.7425 (14)	156
N4—H4⋯O1^ii^	0.88	2.30	2.9779 (16)	134
N4—H4⋯O3^ii^	0.88	2.07	2.8326 (18)	145
C5—H5⋯O3^iii^	0.95	2.46	3.3915 (19)	166
C6—H6⋯O2^iv^	0.95	2.54	3.278 (2)	135

Symmetry codes: (ii) 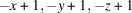; (iii) 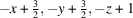; (iv) 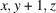.

**Table 3:** Experimental details

Crystal data	
Chemical formula	[Zn(C_9_H_8_N_2_)_4_](NO_3_)_2_
*M*_r_	766.08
Crystal system, space group	Monoclinic, *C*2/*c*
Temperature (K)	100
*a*, *b*, *c* (Å)	20.2757 (5), 8.5411 (2), 20.2328 (5)
β (°)	94.908 (1)
*V* (Å^3^)	3491.00 (15)
*Z*	4
Radiation type	Mo *K*α
μ (mm^−1^)	0.77
Crystal size (mm)	0.33 × 0.28 × 0.21

Data collection	
Diffractometer	Bruker D8 Venture Photon CCD area detector
Absorption correction	Multi-scan (*SADABS*; Krause *et al.*, 2015 ▸)
*T*_min_, *T*_max_	0.676, 0.746
No. of measured, independent and observed [*I* > 2σ(*I*)] reflections	37634, 4362, 3778
*R*_int_	0.039
(sin θ/λ)_max_ (Å^−1^)	0.668

Refinement	
*R*[*F*^2^ > 2σ(*F*^2^)], *wR*(*F*^2^), *S*	0.032, 0.083, 1.08
No. of reflections	4131
No. of parameters	240
H-atom treatment	H-atom parameters constrained
Δρ_max_, Δρ_min_ (e Å^−3^)	0.58, −0.54

Computer programs: *APEX3* and *SAINT* (Bruker, 2016 ▸), *OLEX2.solve* (Bourhis *et al.*, 2015 ▸), *SHELXL2018/3* (Sheldrick, 2015 ▸) and *OLEX2* (Dolomanov *et al.*, 2009 ▸).

## supplementary crystallographic information

### Tetrakis(5-phenyl-1H-imidazole-κN3)zinc(II) dinitrate Crystal data

**Table:**

[Zn(C_9_H_8_N_2_)_4_](NO_3_)_2_	*F*(000) = 1584
*M**_r_* = 766.08	*D*_x_ = 1.458 Mg m^−^^3^
Monoclinic, *C*2/*c*	Mo *K*α radiation, λ = 0.71073 Å
*a* = 20.2757 (5) Å	Cell parameters from 9886 reflections
*b* = 8.5411 (2) Å	θ = 3.9–28.3°
*c* = 20.2328 (5) Å	µ = 0.77 mm^−^^1^
β = 94.908 (1)°	*T* = 100 K
*V* = 3491.00 (15) Å^3^	Block, colourless
*Z* = 4	0.33 × 0.28 × 0.21 mm

### Tetrakis(5-phenyl-1H-imidazole-κN3)zinc(II) dinitrate Data collection

**Table:**

Bruker D8 Venture Photon CCD area detector diffractometer	3778 reflections with *I* > 2σ(*I*)
Graphite monochromator	*R*_int_ = 0.039
ω scans	θ_max_ = 28.3°, θ_min_ = 3.9°
Absorption correction: multi-scan (SADABS; Krause *et al.*, 2015)	*h* = −26→26
*T*_min_ = 0.676, *T*_max_ = 0.746	*k* = −11→11
37634 measured reflections	*l* = −26→26
4362 independent reflections	

### Tetrakis(5-phenyl-1H-imidazole-κN3)zinc(II) dinitrate Refinement

**Table:**

Refinement on *F*^2^	Primary atom site location: iterative
Least-squares matrix: full	Hydrogen site location: inferred from neighbouring sites
*R*[*F*^2^ > 2σ(*F*^2^)] = 0.032	H-atom parameters constrained
*wR*(*F*^2^) = 0.083	*w* = 1/[σ^2^(*F*_o_^2^) + (0.0364*P*)^2^ + 3.5607*P*] where *P* = (*F*_o_^2^ + 2*F*_c_^2^)/3
*S* = 1.08	(Δ/σ)_max_ < 0.001
4131 reflections	Δρ_max_ = 0.58 e Å^−^^3^
240 parameters	Δρ_min_ = −0.54 e Å^−^^3^
0 restraints	

### Tetrakis(5-phenyl-1H-imidazole-κN3)zinc(II) dinitrate Special details

**Table:**

Geometry. All esds (except the esd in the dihedral angle between two l.s. planes) are estimated using the full covariance matrix. The cell esds are taken into account individually in the estimation of esds in distances, angles and torsion angles; correlations between esds in cell parameters are only used when they are defined by crystal symmetry. An approximate (isotropic) treatment of cell esds is used for estimating esds involving l.s. planes.

### Tetrakis(5-phenyl-1H-imidazole-κN3)zinc(II) dinitrate Fractional atomic coordinates and isotropic or equivalent isotropic displacement parameters (Å^2^)

**Table:**

	*x*	*y*	*z*	*U*_iso_*/*U*_eq_	
Zn1	0.500000	0.49768 (2)	0.250000	0.01768 (9)	
N1	0.53202 (5)	0.63867 (13)	0.32410 (5)	0.0197 (2)	
N2	0.59820 (5)	0.73921 (13)	0.40463 (5)	0.0200 (2)	
H2	0.631233	0.748065	0.435560	0.024*	
N3	0.42707 (6)	0.36069 (13)	0.27471 (5)	0.0200 (2)	
N4	0.34930 (6)	0.27794 (14)	0.33445 (6)	0.0259 (3)	
H4	0.319267	0.278378	0.363381	0.031*	
C1	0.58505 (7)	0.61404 (16)	0.36617 (6)	0.0207 (3)	
H1	0.610201	0.520057	0.368544	0.025*	
C2	0.51038 (6)	0.78840 (15)	0.33734 (6)	0.0193 (2)	
H2A	0.473079	0.838709	0.315000	0.023*	
C3	0.55109 (6)	0.85247 (15)	0.38757 (6)	0.0180 (2)	
C4	0.55155 (7)	1.00688 (14)	0.41913 (6)	0.0181 (2)	
C5	0.61083 (7)	1.07279 (17)	0.44644 (7)	0.0243 (3)	
H5	0.651257	1.017424	0.444524	0.029*	
C6	0.61072 (8)	1.21885 (17)	0.47632 (8)	0.0295 (3)	
H6	0.651080	1.262996	0.495073	0.035*	
C7	0.55174 (8)	1.30109 (17)	0.47897 (8)	0.0291 (3)	
H7	0.551861	1.401404	0.499269	0.035*	
C8	0.49274 (7)	1.23615 (17)	0.45189 (7)	0.0255 (3)	
H8	0.452460	1.292215	0.453632	0.031*	
C9	0.49242 (6)	1.08976 (16)	0.42230 (6)	0.0211 (3)	
H9	0.451869	1.045539	0.404097	0.025*	
C10	0.38769 (7)	0.39837 (16)	0.32101 (7)	0.0243 (3)	
H10	0.386876	0.497897	0.341889	0.029*	
C11	0.41252 (6)	0.20659 (15)	0.25808 (6)	0.0198 (2)	
H11	0.432820	0.147352	0.225633	0.024*	
C12	0.36429 (6)	0.15292 (15)	0.29572 (6)	0.0190 (2)	
C13	0.33328 (6)	−0.00064 (14)	0.30077 (7)	0.0186 (3)	
C14	0.33421 (6)	−0.10875 (16)	0.24892 (7)	0.0218 (3)	
H14	0.354879	−0.082393	0.210011	0.026*	
C15	0.30492 (7)	−0.25450 (17)	0.25434 (7)	0.0252 (3)	
H15	0.305487	−0.327515	0.218969	0.030*	
C16	0.27481 (7)	−0.29434 (17)	0.31107 (7)	0.0262 (3)	
H16	0.255042	−0.394519	0.314562	0.031*	
C17	0.27360 (7)	−0.18784 (18)	0.36261 (7)	0.0276 (3)	
H17	0.252828	−0.215062	0.401350	0.033*	
C18	0.30266 (7)	−0.04139 (18)	0.35785 (7)	0.0242 (3)	
H18	0.301760	0.031187	0.393345	0.029*	
O1	0.67549 (5)	0.73031 (12)	0.52263 (5)	0.0244 (2)	
O2	0.71165 (7)	0.51909 (14)	0.48003 (8)	0.0457 (4)	
O3	0.74061 (6)	0.5820 (2)	0.58356 (6)	0.0526 (4)	
N5	0.70964 (6)	0.60692 (15)	0.52796 (6)	0.0258 (3)	

### Tetrakis(5-phenyl-1H-imidazole-κN3)zinc(II) dinitrate Atomic displacement parameters (Å^2^)

**Table:**

	*U*^11^	*U*^22^	*U*^33^	*U*^12^	*U*^13^	*U*^23^
Zn1	0.01953 (12)	0.01818 (12)	0.01528 (12)	0.000	0.00119 (8)	0.000
N1	0.0211 (5)	0.0212 (5)	0.0165 (5)	0.0007 (4)	0.0007 (4)	−0.0006 (4)
N2	0.0191 (5)	0.0229 (5)	0.0175 (5)	0.0024 (4)	−0.0015 (4)	−0.0007 (4)
N3	0.0213 (5)	0.0199 (5)	0.0190 (5)	0.0001 (4)	0.0016 (4)	0.0005 (4)
N4	0.0234 (6)	0.0241 (6)	0.0320 (6)	−0.0023 (4)	0.0123 (5)	−0.0067 (5)
C1	0.0220 (6)	0.0218 (6)	0.0182 (6)	0.0025 (5)	0.0013 (5)	0.0002 (5)
C2	0.0190 (6)	0.0220 (6)	0.0169 (6)	0.0023 (5)	0.0009 (4)	0.0007 (5)
C3	0.0172 (5)	0.0218 (6)	0.0153 (5)	0.0011 (5)	0.0028 (4)	0.0014 (5)
C4	0.0198 (6)	0.0205 (6)	0.0141 (5)	0.0000 (4)	0.0024 (4)	0.0016 (4)
C5	0.0200 (6)	0.0241 (7)	0.0287 (7)	−0.0002 (5)	0.0021 (5)	−0.0006 (5)
C6	0.0274 (7)	0.0246 (7)	0.0359 (8)	−0.0054 (6)	−0.0001 (6)	−0.0027 (6)
C7	0.0381 (8)	0.0195 (6)	0.0300 (7)	0.0001 (6)	0.0039 (6)	−0.0025 (5)
C8	0.0281 (7)	0.0257 (7)	0.0233 (6)	0.0072 (5)	0.0051 (5)	0.0017 (5)
C9	0.0200 (6)	0.0265 (7)	0.0169 (6)	0.0017 (5)	0.0012 (4)	0.0012 (5)
C10	0.0232 (6)	0.0202 (6)	0.0302 (7)	−0.0007 (5)	0.0062 (5)	−0.0043 (5)
C11	0.0207 (6)	0.0207 (6)	0.0181 (6)	−0.0005 (5)	0.0020 (5)	−0.0018 (5)
C12	0.0171 (5)	0.0206 (6)	0.0190 (6)	0.0014 (5)	0.0000 (4)	−0.0020 (5)
C13	0.0153 (6)	0.0199 (6)	0.0201 (6)	0.0004 (4)	−0.0004 (5)	0.0006 (5)
C14	0.0187 (6)	0.0237 (6)	0.0231 (6)	0.0024 (5)	0.0024 (5)	−0.0015 (5)
C15	0.0236 (6)	0.0226 (6)	0.0287 (7)	0.0006 (5)	−0.0009 (5)	−0.0059 (5)
C16	0.0253 (7)	0.0212 (6)	0.0311 (7)	−0.0039 (5)	−0.0038 (5)	0.0023 (6)
C17	0.0287 (7)	0.0310 (7)	0.0230 (7)	−0.0067 (6)	0.0013 (5)	0.0033 (6)
C18	0.0256 (7)	0.0267 (7)	0.0203 (6)	−0.0042 (5)	0.0022 (5)	−0.0021 (5)
O1	0.0228 (5)	0.0292 (5)	0.0207 (5)	0.0060 (4)	−0.0011 (4)	−0.0020 (4)
O2	0.0471 (8)	0.0277 (6)	0.0664 (9)	−0.0079 (5)	0.0281 (7)	−0.0178 (6)
O3	0.0389 (7)	0.0844 (11)	0.0365 (7)	0.0302 (7)	0.0154 (5)	0.0324 (7)
N5	0.0219 (5)	0.0271 (6)	0.0299 (6)	0.0007 (5)	0.0111 (5)	0.0059 (5)

### Tetrakis(5-phenyl-1H-imidazole-κN3)zinc(II) dinitrate Geometric parameters (Å, º)

**Table:**

Zn1—N1^i^	1.9881 (11)	C7—H7	0.9500
Zn1—N1	1.9882 (11)	C7—C8	1.388 (2)
Zn1—N3^i^	1.9829 (11)	C8—H8	0.9500
Zn1—N3	1.9828 (11)	C8—C9	1.386 (2)
N1—C1	1.3297 (17)	C9—H9	0.9500
N1—C2	1.3856 (17)	C10—H10	0.9500
N2—H2	0.8800	C11—H11	0.9500
N2—C1	1.3357 (17)	C11—C12	1.3689 (18)
N2—C3	1.3824 (16)	C12—C13	1.4618 (17)
N3—C10	1.3217 (18)	C13—C14	1.3990 (19)
N3—C11	1.3841 (17)	C13—C18	1.4012 (19)
N4—H4	0.8800	C14—H14	0.9500
N4—C10	1.3320 (18)	C14—C15	1.388 (2)
N4—C12	1.3739 (17)	C15—H15	0.9500
C1—H1	0.9500	C15—C16	1.388 (2)
C2—H2A	0.9500	C16—H16	0.9500
C2—C3	1.3675 (18)	C16—C17	1.386 (2)
C3—C4	1.4649 (18)	C17—H17	0.9500
C4—C5	1.3975 (19)	C17—C18	1.389 (2)
C4—C9	1.3985 (18)	C18—H18	0.9500
C5—H5	0.9500	O1—N5	1.2606 (16)
C5—C6	1.386 (2)	O2—N5	1.2295 (19)
C6—H6	0.9500	O3—N5	1.2585 (18)
C6—C7	1.392 (2)		
			
N1^i^—Zn1—N1	105.44 (6)	C8—C7—H7	120.1
N3—Zn1—N1^i^	110.19 (4)	C7—C8—H8	119.9
N3—Zn1—N1	111.70 (4)	C9—C8—C7	120.20 (13)
N3^i^—Zn1—N1	110.18 (4)	C9—C8—H8	119.9
N3^i^—Zn1—N1^i^	111.70 (4)	C4—C9—H9	119.9
N3—Zn1—N3^i^	107.68 (7)	C8—C9—C4	120.27 (13)
C1—N1—Zn1	125.51 (9)	C8—C9—H9	119.9
C1—N1—C2	105.92 (11)	N3—C10—N4	110.84 (12)
C2—N1—Zn1	128.06 (9)	N3—C10—H10	124.6
C1—N2—H2	125.8	N4—C10—H10	124.6
C1—N2—C3	108.39 (11)	N3—C11—H11	125.3
C3—N2—H2	125.8	C12—C11—N3	109.31 (11)
C10—N3—Zn1	122.94 (9)	C12—C11—H11	125.3
C10—N3—C11	105.94 (11)	N4—C12—C13	122.68 (12)
C11—N3—Zn1	130.38 (9)	C11—C12—N4	105.10 (11)
C10—N4—H4	125.6	C11—C12—C13	132.17 (12)
C10—N4—C12	108.79 (11)	C14—C13—C12	120.42 (12)
C12—N4—H4	125.6	C14—C13—C18	119.31 (12)
N1—C1—N2	110.88 (12)	C18—C13—C12	120.27 (12)
N1—C1—H1	124.6	C13—C14—H14	120.0
N2—C1—H1	124.6	C15—C14—C13	119.98 (12)
N1—C2—H2A	125.3	C15—C14—H14	120.0
C3—C2—N1	109.40 (11)	C14—C15—H15	119.8
C3—C2—H2A	125.3	C14—C15—C16	120.45 (13)
N2—C3—C4	122.87 (11)	C16—C15—H15	119.8
C2—C3—N2	105.41 (11)	C15—C16—H16	120.0
C2—C3—C4	131.70 (12)	C17—C16—C15	119.92 (13)
C5—C4—C3	120.55 (12)	C17—C16—H16	120.0
C5—C4—C9	119.31 (12)	C16—C17—H17	119.9
C9—C4—C3	120.14 (12)	C16—C17—C18	120.27 (13)
C4—C5—H5	119.9	C18—C17—H17	119.9
C6—C5—C4	120.10 (13)	C13—C18—H18	120.0
C6—C5—H5	119.9	C17—C18—C13	120.07 (13)
C5—C6—H6	119.9	C17—C18—H18	120.0
C5—C6—C7	120.28 (14)	O2—N5—O1	119.84 (14)
C7—C6—H6	119.9	O2—N5—O3	123.65 (14)
C6—C7—H7	120.1	O3—N5—O1	116.51 (13)
C8—C7—C6	119.83 (13)		
			
Zn1—N1—C1—N2	171.98 (9)	C4—C5—C6—C7	−0.4 (2)
Zn1—N1—C2—C3	−172.01 (9)	C5—C4—C9—C8	0.38 (19)
Zn1—N3—C10—N4	−171.23 (9)	C5—C6—C7—C8	0.3 (2)
Zn1—N3—C11—C12	169.66 (9)	C6—C7—C8—C9	0.1 (2)
N1—C2—C3—N2	0.22 (14)	C7—C8—C9—C4	−0.4 (2)
N1—C2—C3—C4	178.14 (12)	C9—C4—C5—C6	0.0 (2)
N2—C3—C4—C5	26.89 (19)	C10—N3—C11—C12	−0.57 (15)
N2—C3—C4—C9	−152.98 (12)	C10—N4—C12—C11	−1.02 (16)
N3—C11—C12—N4	0.97 (15)	C10—N4—C12—C13	176.80 (12)
N3—C11—C12—C13	−176.56 (13)	C11—N3—C10—N4	−0.09 (16)
N4—C12—C13—C14	160.74 (13)	C11—C12—C13—C14	−22.1 (2)
N4—C12—C13—C18	−19.7 (2)	C11—C12—C13—C18	157.50 (15)
C1—N1—C2—C3	0.08 (14)	C12—N4—C10—N3	0.71 (17)
C1—N2—C3—C2	−0.44 (14)	C12—C13—C14—C15	179.68 (12)
C1—N2—C3—C4	−178.59 (12)	C12—C13—C18—C17	−179.60 (13)
C2—N1—C1—N2	−0.37 (15)	C13—C14—C15—C16	−0.2 (2)
C2—C3—C4—C5	−150.72 (14)	C14—C13—C18—C17	0.0 (2)
C2—C3—C4—C9	29.4 (2)	C14—C15—C16—C17	0.3 (2)
C3—N2—C1—N1	0.52 (15)	C15—C16—C17—C18	−0.2 (2)
C3—C4—C5—C6	−179.86 (13)	C16—C17—C18—C13	0.1 (2)
C3—C4—C9—C8	−179.74 (12)	C18—C13—C14—C15	0.1 (2)

Symmetry code: (i) −*x*+1, *y*, −*z*+1/2.

### Tetrakis(5-phenyl-1H-imidazole-κN3)zinc(II) dinitrate Hydrogen-bond geometry (Å, º)

**Table:**

*D*—H···*A*	*D*—H	H···*A*	*D*···*A*	*D*—H···*A*
N2—H2···O1	0.88	1.91	2.7425 (14)	156
N4—H4···O1^ii^	0.88	2.30	2.9779 (16)	134
N4—H4···O3^ii^	0.88	2.07	2.8326 (18)	145
C5—H5···O3^iii^	0.95	2.46	3.3915 (19)	166
C6—H6···O2^iv^	0.95	2.54	3.278 (2)	135

Symmetry codes: (ii) −*x*+1, −*y*+1, −*z*+1; (iii) −*x*+3/2, −*y*+3/2, −*z*+1; (iv) *x*, *y*+1, *z*.
